# Altering the Mitochondrial Fatty Acid Synthesis (mtFASII) Pathway Modulates Cellular Metabolic States and Bioactive Lipid Profiles as Revealed by Metabolomic Profiling

**DOI:** 10.1371/journal.pone.0151171

**Published:** 2016-03-10

**Authors:** Hayley B. Clay, Angelika K. Parl, Sabrina L. Mitchell, Larry Singh, Lauren N. Bell, Deborah G. Murdock

**Affiliations:** 1 Center for Human Genetics Research, Department of Molecular Physiology and Biophysics, Vanderbilt University, Nashville, Tennessee, United States of America; 2 Neuroscience Graduate Program, Vanderbilt University, Nashville, Tennessee, United States of America; 3 Center for Mitochondrial and Epigenomic Medicine, Children’s Hospital of Philadelphia, Philadelphia, Pennsylvania, United States of America; 4 Metabolon, Incorporated, Durham, North Carolina, United States of America; East Tennessee State University, UNITED STATES

## Abstract

Despite the presence of a cytosolic fatty acid synthesis pathway, mitochondria have retained their own means of creating fatty acids via the mitochondrial fatty acid synthesis (mtFASII) pathway. The reason for its conservation has not yet been elucidated. Therefore, to better understand the role of mtFASII in the cell, we used thin layer chromatography to characterize the contribution of the mtFASII pathway to the fatty acid composition of selected mitochondrial lipids. Next, we performed metabolomic analysis on HeLa cells in which the mtFASII pathway was either hypofunctional (through knockdown of mitochondrial acyl carrier protein, ACP) or hyperfunctional (through overexpression of mitochondrial enoyl-CoA reductase, MECR). Our results indicate that the mtFASII pathway contributes little to the fatty acid composition of mitochondrial lipid species examined. Additionally, loss of mtFASII function results in changes in biochemical pathways suggesting alterations in glucose utilization and redox state. Interestingly, levels of bioactive lipids, including lysophospholipids and sphingolipids, directly correlate with mtFASII function, indicating that mtFASII may be involved in the regulation of bioactive lipid levels. Regulation of bioactive lipid levels by mtFASII implicates the pathway as a mediator of intracellular signaling.

## Introduction

**Fig 1:**
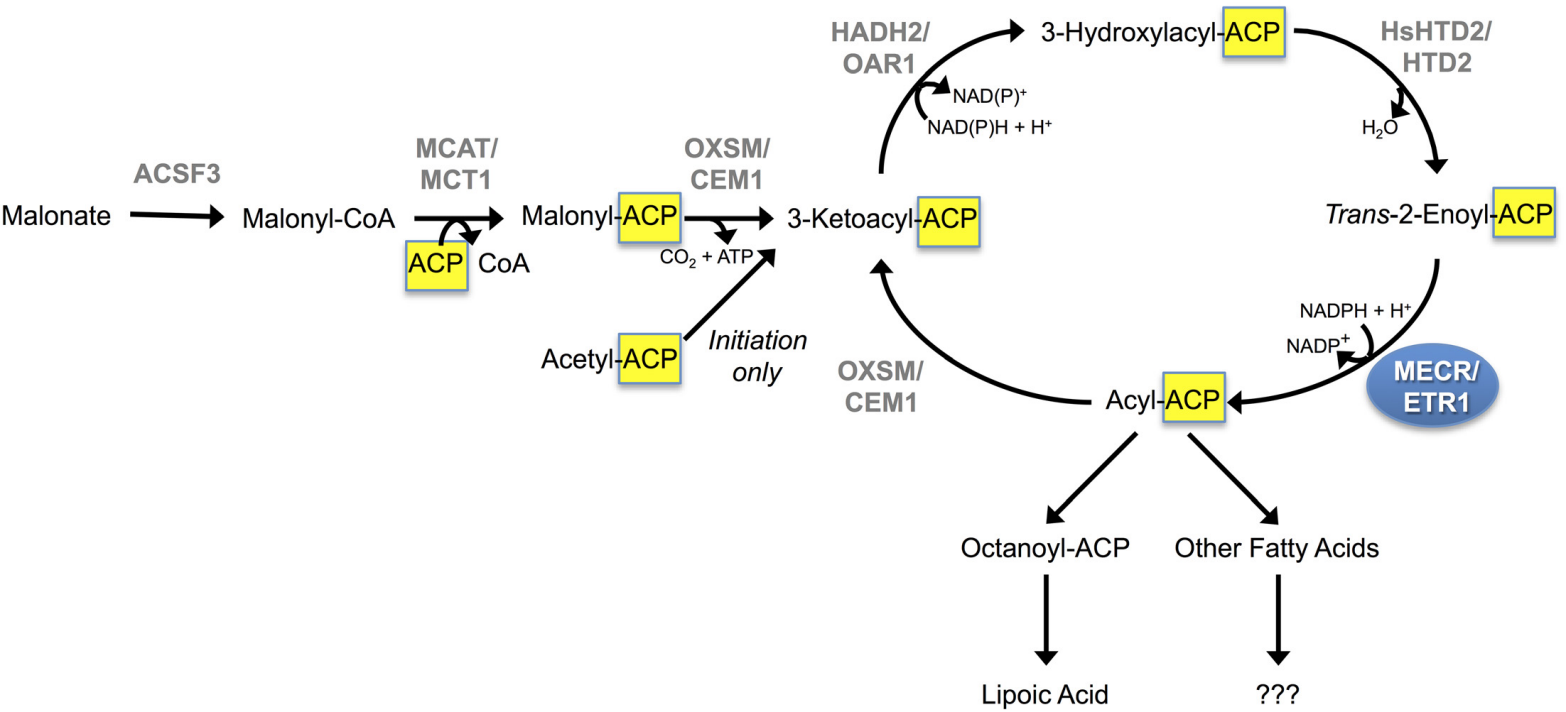
The mtFASII pathway. In the mitochondria, ACSF3 links malonate to CoA, producing malonyl-CoA. MCAT then transfers the malonyl moiety from malonyl-CoA onto ACP, resulting in malonyl-ACP. KAS performs the initial condensation reaction of the mtFASII pathway, condensing malonyl ACP with acetyl-CoA, acetyl-ACP, or acyl-ACP derived from previous mtFASII cycles. The initial condensation reaction produces 3-ketoacyl-ACP. KAR then reduces 3-ketoacyl-ACP, producing 3-hydroxyacyl-ACP. HsHTD2 then dehydrates 3-hydroxyacyl-ACP to *trans*-2,3-enoyl-ACP. Finally, MECR reduces *trans*-2,3-enoyl-ACP to acyl-ACP. Octanoyl-ACP is shuttled into the lipoic acid synthesis pathway, but other uses of mtFASII products are unknown.

Mitochondria are cellular organelles with a bacterial evolutionary lineage. Despite the time since their last common ancestor, mitochondria retain many bacterial characteristics. One conserved, bacteria-like feature of mitochondria is their fatty acid synthesis (mtFASII) pathway (Fig 1) [1–3]. Similar to the bacterial fatty acid synthesis pathway, mtFASII synthesizes fatty acids using a series of enzymes, whereas the eukaryotic cytosolic system for fatty acid synthesis (FASI) uses a single multifunctional enzyme, fatty acid synthase. In light of the presence of FASI, the reason for the conservation of the mitochondrial pathway is unknown. Likewise, the complete identities and uses of mtFASII products in mammalian cells are not yet known.

In the mitochondria, fatty acids are synthesized from the precursor molecules malonate, malonyl-CoA, and acetyl-CoA, and their elongation into fatty acids requires ATP and NADPH [4, 5]. The mtFASII pathway is capable of synthesizing fatty acids with acyl chains of at least 14 carbons long (myristic acid) in mammalian cells, and in other species, mtFASII can synthesize fatty acids of at least 16 carbons in length (palmitic acid) [6–8]. The one known destination of mtFASII products is in the creation of lipoic acid. To create lipoic acid, lipoyl synthase uses octanoic acid from the mtFASII pathway and S-adenosyl methionine [7, 9]. Lipoic acid serves as a cofactor for many enzymes, including pyruvate dehydrogenase, α-ketoglutarate dehydrogenase, and the branched chain oxoacid dehydrogenase. Therefore, knockdown of mtFASII components results in reduced cellular lipoic acid content and protein lipoylation levels [10, 11]. Application of exogenous lipoate does not alleviate the effects of mtFASII knockdown on protein lipoylation, indicating that a mitochondrial origin of fatty acids may be required for lipoylation to occur [12].

Whether through the direct impact of the fatty acids produced, downstream consequences of fatty acid synthesis, or dual roles of mtFASII enzymes, the mtFASII pathway is important for maintaining mitochondrial health and function. Expression of mtFASII proteins in mammals correlates by tissue with mitochondrial activity, and loss of any mtFASII enzyme in mammals or yeast results in mitochondrial dysfunction [3, 10, 13]. Alteration of mtFASII function by deletion or knockdown of its components results in respiratory deficiency [11, 12, 14–17], increased reactive oxygen species (ROS) [12], rudimentary mitochondria with abnormal morphology [13, 15], and slowed cell growth [12, 15]. In addition, deletion of any mtFASII component in yeast results in impaired mitochondrial tRNA processing by mitochondrial RNaseP [18, 19].

Acyl carrier protein (ACP) is integral to mtFASII as the mode of transport for nascent fatty acids between the mtFASII enzymes (Fig 1). To begin the mammalian mtFASII pathway, malonate is transferred to CoA by the malonyl-CoA synthetase (ACSF3), [10] and then to ACP by malonyltransferase (MCAT) [4, 20–22]. Fatty acids remain bound to ACP by a thioester bond throughout chain elongation. While ACP has been identified as a component of complex I of the electron transport chain, the majority of ACP is found in soluble form in the mitochondrial matrix [22, 23].

Mitochondrial enoyl-CoA reductase (MECR), the last enzyme in the mtFASII pathway (Fig 1), is a 2-enoyl thioester reductase that acts as a dimer, with a pocket forming between the two monomers that can accommodate fatty acid chains up to 16 carbons in length [1, 13]. Upregulation of MECR has been shown to cause activation of the PPARα transcription pathway, either through its role as a coactivator [24] or through increased mtFASII activity [25].

Given the lingering questions concerning the role of mtFASII in the cell, we sought to identify potential functions of mammalian mtFASII through knockdown of ACP (ACP KD), and by promoting mtFASII function through MECR overexpression (MECR OX) in HeLa cells. Here, we demonstrate that the mtFASII pathway contributes little, if anything, to the fatty acid composition of selected mitochondrial lipids. Metabolomics analysis of ACP KD and MECR OX cells revealed the identities of biochemicals altered by mtFASII loss or upregulation, providing insight into the important roles this pathway plays in the cell. We show that altering the mtFASII pathway causes corresponding changes in cellular metabolic state, and a shift between glycolysis and anaerobic respiration. Additionally, manipulation of the mtFASII pathway alters cellular levels of bioactive lipids, including lysophospholipids and sphingolipids, pointing to a possible role for mtFASII in lipid signaling and remodeling.

## Materials and Methods

### shRNA knockdown of ACP

#### Cell culture conditions and siRNA-mediated RNA knockdown

All cell cultures were maintained at 37°C and 5% CO_2_. HeLa cells were plated in 10-cm cell culture dishes (CellTreat) at a density of 1.5 x 10^5^ cells/mL in DMEM + 10% FBS (Mediatech, Gibco). Knockdown of the mtFASII pathway was achieved using Qiagen Flexitube siRNAs specific for the gene for ACP (*NDUFAB1*) as described in Parl et al., 2013[25]. Control cells were transfected with Allstars negative control siRNA (Qiagen). Additionally, as a negative control cells were transfected with siRNAs specific for *METTL9*, a nuclear gene encoding a mitochondrial protein, HeLa cells were transfected with siRNA using HiPerfect Transfection Reagent (Qiagen). In order to maintain the knockdown, cells were re-transfected after 48 h: cells were trypsinized, resuspended in double their original volume of DMEM + 10% FBS, then plated in a 15-cm dish for an additional 48 h. At 96 h, cells were harvested by trypsinization and centrifugation. Knockdown efficiency was measured after 96 h using real time quantitative RT-PCR. Cell viability was unaltered in the siRNA treated cells as determined by cell count and trypan blue uptake.

### Overexpression of MECR

#### Plasmid construction

The MECR OX plasmid was constructed as described in Parl et al., 2013[25].

#### Cell culture conditions and plasmid-mediated MECR overexpression

All cell cultures were maintained at 37°C and 5% CO_2_. HeLa cells were plated in 10-cm cell culture dishes (CellTreat) at a density of 1.5 x 10^5^ cells/mL in DMEM + 10% FBS (Mediatech, Gibco). After 24 h, MECR OX was achieved by transfecting cells with the pSG5 vector with cloned-in MECR under SV40 promoter control. Control cells received an equal amount of empty pSG5 vector (Agilent Technologies, Inc). HeLa cells were transfected using FuGENE transfection reagent (Promega). After an additional 24 h, cells were harvested by trypsinization and centrifugation. MECR OX was confirmed using real-time quantitative RT-PCR. Cell viability was unaltered in the MECR OX cells as determined by cell count and trypan blue uptake.

### Real time quantitative RT-PCR

Total RNA was isolated using TRIzol Reagent (Life Technologies) according to manufacturer’s protocols. First-strand cDNA was created from total RNA using SuperScript^®^ III First-Strand Synthesis SuperMix for qRT-PCR (Life Technologies). Quantitative RT-PCR was performed using TaqMan Expression Assays (Life Technologies) on the ABI 7900 platform according to manufacturer’s protocols.

### Western blotting

Whole-cell protein was isolated using RIPA buffer according to manufacturer’s protocol (Sigma-Aldrich). Protein was then blotted using 12% Bio-Rad precast gels and PVDF membranes. To indicate protein lipoylation levels, an anti-lipoate antibody was used. To detect levels of electron transport chain proteins, an OXPHOS antibody cocktail (MitoSciences MS604) was used. To indicate the presence of ER contamination in isolated mitochondria samples, an antibody against FACLS4 was used (Santa Cruz), and an anti-PDH antibody (Santa Cruz) was used for protein normalization. These blots indicated that mitochondrial samples were free from ER contamination (data not shown).

### Mitochondrial isolation for thin layer chromatography

Mitochondria from HeLa cells were harvested using the MACS Mitochondria Isolation Kit (Miltenyi) according to manufacturer’s protocol. Briefly, approximately 10^7^ HeLa cells were washed and lysed, then incubated with magnetic beads linked to an anti-TOM22 antibody, which binds the outer surface of the mitochondria. The mitochondria-linked beads were passed through a column in a magnetic field, retaining the beads and mitochondria. Upon removal from the magnetic field, isolated mitochondria were then flushed from the column.

### Citrate Synthase Assay

Mitochondria isolated using the MACS Mitochondria Isolation Kit (Miltenyi) were examined for intactness using a citrate synthase assay kit (Sigma-Aldrich) according to manufacturer’s instructions. Briefly, isolated mitochondria from approximately 10^7^ HeLa cells were incubated at 37°C for 0 minutes or 6 hours, then were separated into two equal aliquots, one in which mitochondria were left intact, and one in which mitochondria were lysed. Because citrate synthase activity is restricted to the mitochondrial matrix, inner mitochondrial membrane intactness can be assessed by comparing the citrate synthase activity in a sample of intact mitochondria to that of a sample of lysed mitochondria using an indicator dye detected spectrophotometrically. This mitochondrial intactness assay indicated that, after 6 hours of incubation in mitochondrial secretion assay buffer, the percent of mitochondria that were ruptured rose from 19.0% at 0 minutes to 29.5% at 6 hours (data not shown). These data indicate that mitochondria isolated in our laboratory are largely intact, even after several hours.

### Thin layer chromatography-gas chromatography

Lipids were extracted using the method of Folch-Lees [26]. Briefly, isolated mitochondria were homogenized in a 2:1 chloroform:methanol solution, followed by addition of 0.2 volumes water. Aqueous and lipophilic phases were then separated by centrifugation, allowing for the isolation of mitochondrial lipids by thin layer chromatography [26]. Individual lipid classes were separated by thin layer chromatography using Silica Gel 60 A plates developed in petroleum ether, ethyl ether, acetic acid (80:20:1) and visualized by rhodamine 6G. Phospholipids were scraped from the plates and methylated using BF_3_ /methanol as previously described [27]. Briefly, BF3/methanol was added to lipids that had been evaporated to dryness, then heated, cooled, and recovered by the addition of pentane and water [27]. The methylated fatty acids were extracted and analyzed by gas chromatography. Gas chromatographic analyses were carried out on an Agilent 7890A gas chromatograph equipped with flame ionization detectors, a capillary column (SP2380, 0.25 mm x 30 m, 0.25 μm film, Supelco, Bellefonte, PA). Helium was used as a carrier gas. The oven temperature was programmed from 160°C to 230°C at 4°C/min. Fatty acid methyl esters were identified by comparing the retention times to those of known standards. Inclusion of dipentadecanoyl phosphatidylcholine (C15:0) in the samples permitted quantitation of total phospholipid in the sample.

### Metabolomics

#### Sample preparation and metabolic profiling

Sample preparation and metabolic profiling was performed on samples of 5 x 10^6^ HeLa cells. The untargeted metabolic profiling platform employed for this analysis was performed by Metabolon (Durham, NC) and combined three independent platforms: ultrahigh performance liquid chromatography/tandem mass spectrometry (UHLC/MS/MS) optimized for basic species, UHLC/MS/MS optimized for acidic species, and gas chromatography/mass spectrometry (GC/MS). Samples were processed as previously described [28–31]. Briefly, cells were homogenized in a minimum volume of water, and 100 μL was withdrawn for subsequent analyses. Using an automated liquid handler (Hamilton LabStar, Salt Lake City, UT), protein was precipitated from the homogenized cells with methanol that contained four standards to report on extraction efficiency. The resulting supernatant was split into equal aliquots for analysis on the three platforms. Aliquots, dried under nitrogen and vacuum-desiccated, were subsequently either reconstituted in 50 μL 0.1% formic acid in water (acidic conditions) or in 50 μL 6.5 mM ammonium bicarbonate in water, pH 8 (basic conditions) for the two UHLC/MS/MS analyses, or derivatized to a final volume of 50 μL for GC/MS analysis using equal parts bistrimethyl-silyl-trifluoroacetamide and solvent mixture acetonitrile:dichloromethane:cyclohexane (5:4:1) with 5% triethylamine at 60°C for one hour. In addition, three types of controls were analyzed in concert with the experimental samples: samples generated from pooled experimental samples served as technical replicates throughout the data set, extracted water samples served as process blanks, and a cocktail of standards spiked into every analyzed sample allowed instrument performance monitoring.

For UHLC/MS/MS analysis, aliquots were separated using a Waters Acquity UPLC (Waters, Millford, MA) and analyzed using an LTQ mass spectrometer (Thermo Fisher Scientific, Inc., Waltham, MA), which consisted of an electrospray ionization (ESI) source and linear ion-trap (LIT) mass analyzer. The MS instrument scanned 99–1000 *m/z* and alternated between MS and MS^2^ scans using dynamic exclusion with approximately 6 scans per second. Derivatized samples for GC/MS were separated on a 5% phenyldimethyl silicone column with helium as the carrier gas and a temperature ramp from 60°C to 340°C, and then analyzed on a Thermo-Finnigan Trace DSQ MS (Thermo Fisher Scientific, Inc.) operated at unit mass resolving power with electron impact ionization and a 50–750 atomic mass unit scan range.

#### Metabolite identification and data analysis

Metabolites were identified by automated comparison of the ion features in the experimental samples to a reference library of chemical standard entries that included retention time, mass-to-charge ratio (*m/z*), preferred adducts, and in-source fragments as well as associated MS spectra, and curated by visual inspection for quality control using software developed at Metabolon [32]. This software allows for the identification and grouping of multiple ions related to a given metabolite, which are then compared against a reference library.

Experimental samples and controls were randomized across a one-day platform run. Any missing values were assumed to be below the limits of detection. Therefore, for statistical analysis and data display purposes, these values were imputed with the compound minimum (minimum value imputation). Statistical analysis of log-transformed data was performed using “R” version 2.14 (http://cran.r-project.org/), which is a freely available, open-source software package. Welch’s t-tests were performed to compare data between experimental groups. Multiple comparisons were accounted for by estimating the false discovery rate (FDR) using q-values [33]. Metabolites were deemed significantly changed when p ≤0.05, and metabolites were considered to have trending changes when 0.05 < p < 0.10.

### Glucose assay

Cell culture media content was assayed using a Beckman glucose analyzer (Beckman Instruments, Fullerton, CA), which is accurate to 450 mg/dL. The assay used was the glucose oxidase method, in which a known amount of glucose oxidase is added to an oxygen-saturated solution with unknown glucose concentration. The rate of oxygen consumption by glucose oxidase is proportional to the solution’s glucose concentration [34]. The reaction sequence is:

$$\beta -D-glucose+O_{2}\;\to \;gluconic\;acid+H_{2}O_{2}$$ (1)

$$H_{2}O_{2}+ethanol\;\to \;acetaldehyde+H_{2}O$$ (2)

$$H_{2}O_{2}+2H^{+}\;+2I{\;}^{-}\to \;I_{2}+H_{2}O$$ (3)

Cell culture media glucose concentrations were determined by comparing the depletion of oxygen levels in cell culture media compared to that of a standard solution. Each sample was measured four times, using 10 μL of sample per reading.

### Lactate assay

Levels of lactate in the medium of ACP KD and MECR OX cells and controls were measured using the Lactate Colorimetric Assay Kit (Biovision) at 96 h (ACP KD) or 24 h (MECR OX) after transfection. At least three independent samples of each cell state (ACP KD, MECR OX, or control) were assayed in triplicate.

### ELISA for sphingosine-1-phosphate

Sphingosine-1-phosphate (S1P) levels were determined in ACP KD cells (96 h) using the S1P ELISA Kit (Echelon Biosciences) according to manufacturer’s protocol. Protein concentrations in each sample were first determined using BSA protein assay (Bio-Rad) according to manufacturer’s protocol. Samples were assayed in triplicate using 40 μg protein per well.

## Results

**Fig 2:**
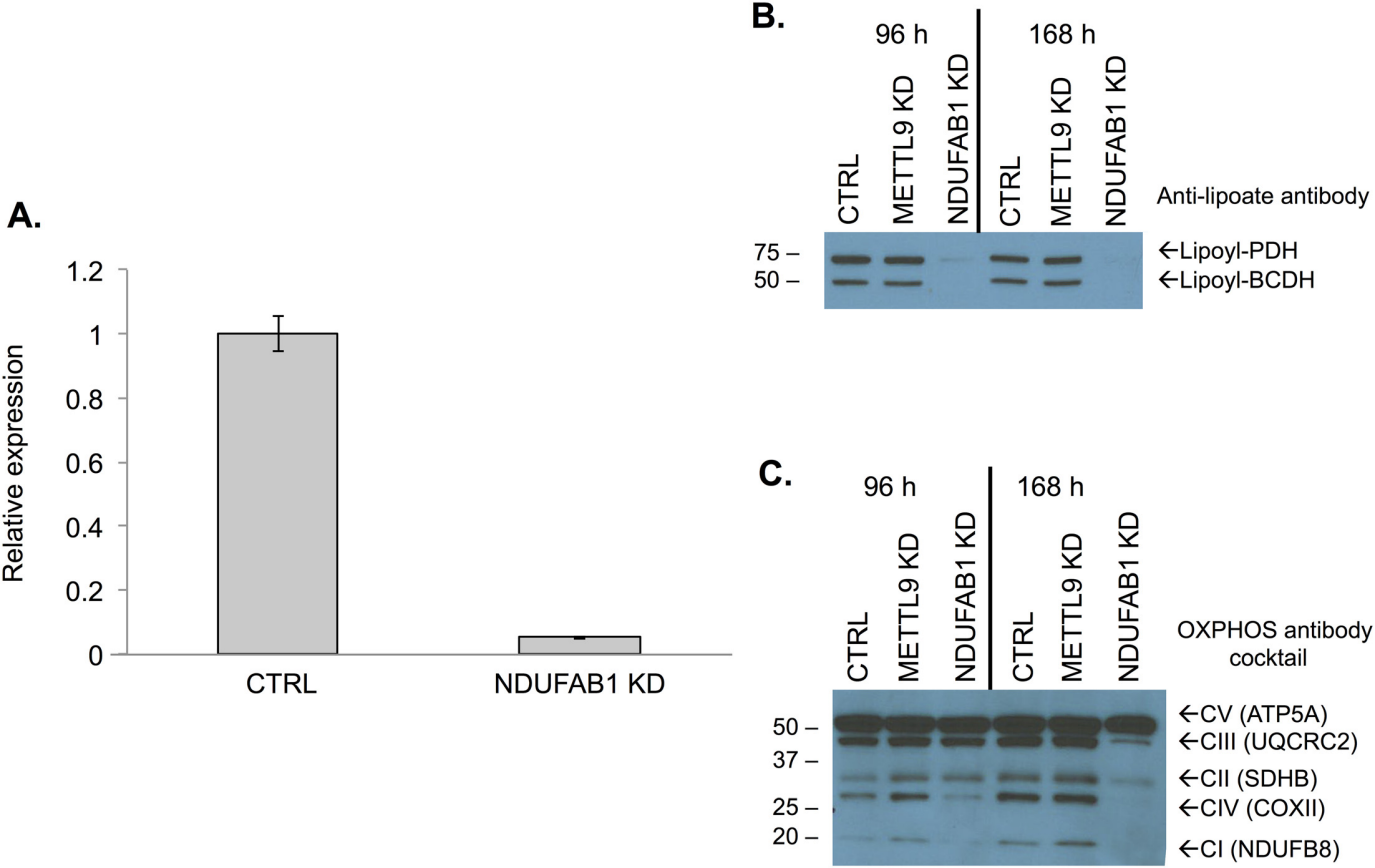
Characterization of ACP KD. A) Relative expression of ACP in control siRNA-treated and ACP siRNA-treated cells as measured by Taqman assay. B) Western blot using anti-lipoate antibody to compare protein from control siRNA-treated, negative control METTL9 siRNA-treated, and ACP siRNA-treated cells at 96 h and 168 h. C) Western blot using OXPHOS antibody cocktail to compare protein from control siRNA-treated, negative control METTL9 siRNA-treated, and ACP siRNA-treated cells at 96 h and 168 h.

**Fig 3:**
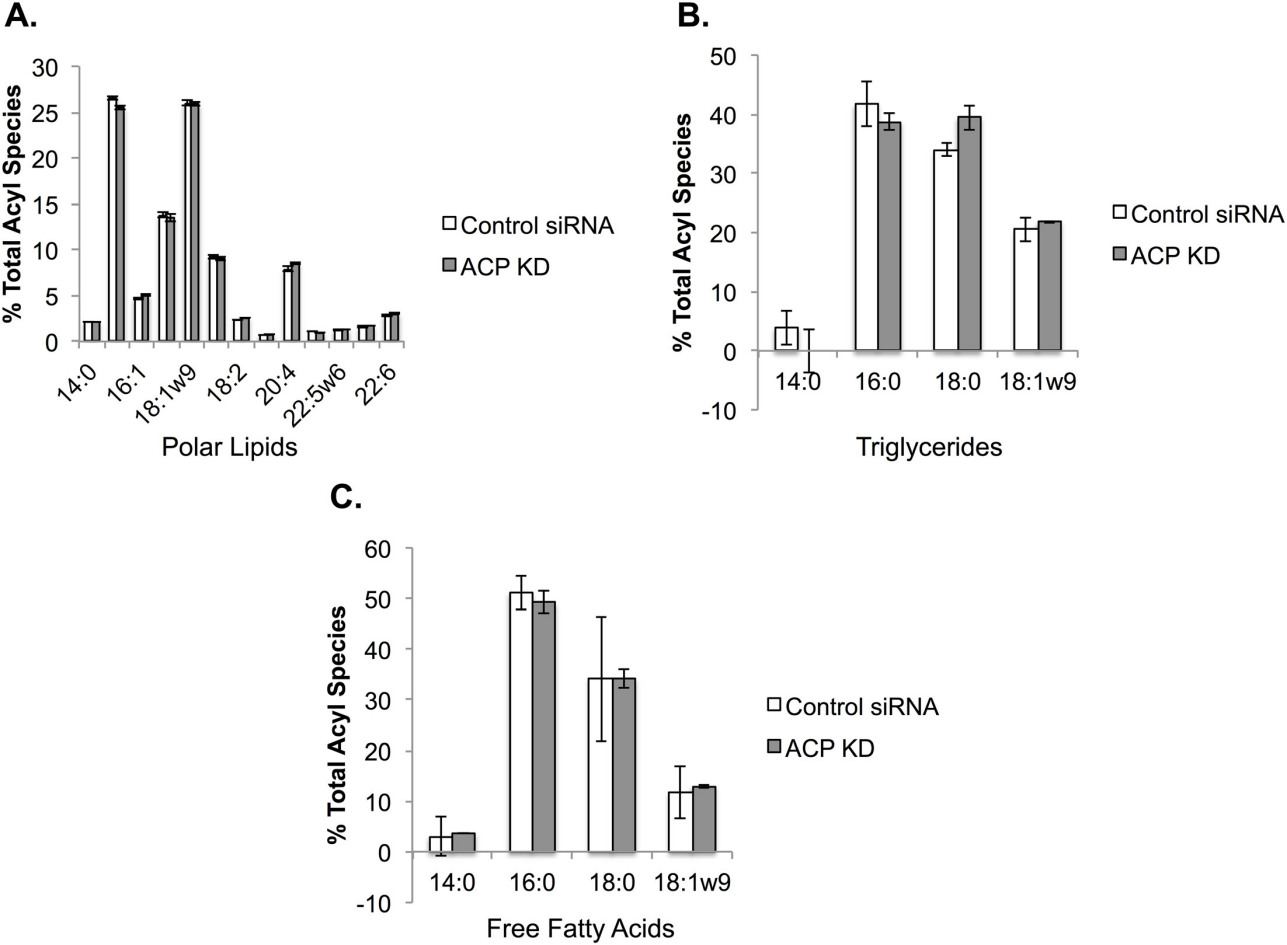
ACP KD does not alter the fatty acid composition of selected mitochondrial lipids. Lipids from isolated mitochondria of control and ACP KD cells were separated into different lipid classes by TLC, and the levels of individual types of fatty acid side chains were determined using gas chromatography. Fatty acid side chain levels are expressed as μg lipid. n = 2, * p < 0.05.

The goal of this study was to explore the cellular role of the fatty acids produced by the mtFASII pathway. To accomplish this, siRNA was used to knockdown the expression of the gene encoding ACP (*NDUFAB1*). Cells treated with NDUFAB1 specific siRNA had less than 10% of the NDUFAB1 mRNA when compared to control cells by real time quantitative PCR (Fig 2A). In addition, knockdown of *NDUFAB1* resulted in several phenotypes reported previously for NDUFAB1 deficiency: defective lipoylation of proteins, and altered levels of several protein components of oxidative phosphorylation(Fig 2B) [7,35]. Western blot analysis with an antibody cocktail for several proteins involved in oxidative phosphorylation showed decreased levels of UQCRC of complex III, MTCOI of complex I, SDH3 of complex II, and NDUFB8 of complex I, but not ATP5A of complex V (Fig 2C). This result is consistent with results from selective loss of OXPHOS enzyme subunits in mtFASII knockout mice [35]. To evaluate the contribution of the mtFASII pathway to the fatty acid profile of mitochondria, lipids were extracted from mitochondria isolated from control and ACP KD cells. Individual lipid classes were isolated by TLC, and the fatty acid side chain lengths determined by gas chromatography. No steady state changes in fatty acid composition were observed in neutral phospholipids, triglycerides, or free fatty acids (Fig 3).

The lack of changes in the major pools of fatty acids in mitochondrial polar lipids, triglycerides, and free fatty acids suggested that determining the role of mtFASII products in the cell required a more agnostic approach. To this end, we performed an untargeted metabolomics study of cells with downregulated or upregulated mtFASII function. Downregulation was achieved by knockdown of the gene encoding ACP, while upregulation was achieved by overexpressing the gene encoding MECR, a system that has been previously characterized [25]. Six independent samples from each experimental condition and their respective controls were extracted and analyzed using a combination of three independent platforms: UHLC/MS/MS optimized for basic species, UHLC/MS/MS optimized for acidic species, and GC/MS. A total of 296 biochemicals were measured by these three independent platforms. A total of 115 biochemicals were significantly altered (p≤0.05) in the ACP KD, and levels of 57 biochemicals were altered in the MECR OX cells (Table 1). Of the 36 biochemicals that were significantly altered in both conditions, 29 of them were altered in opposite directions, and seven were altered in the same direction, supporting the idea that the two model systems are influencing mtFASII in opposite directions. In order to interpret the biochemical changes observed in both experimental conditions, these data were analyzed in the context of biological pathways. Descriptions of these pathways and interpretations of the changes are discussed below.

**Table 1:** Significantly altered biochemicals as measured by metabolomics analysis.

	ACP/Control	MECR/Control
**Total Biochemicals p≤0.05**	115	57
**Biochemicals Increased**	57	33
**Biochemicals Decreased**	58	24
**Total Biochemicals 0.05≤p≤0.10**	28	24
**Biochemicals Increased**	18	12
**Biochemicals Decreased**	10	12

Levels of metabolites from ACP KD cells, MECR OX cells, and their respective controls were analyzed using metabolomics analysis, which included UHLC/MS/MS and GC/MS. The total number of biochemicals that whose levels differed from control significantly for both ACP KD and MECR OX conditions are presented.

### Glycolysis and sorbitol pathways

**Fig 4:**
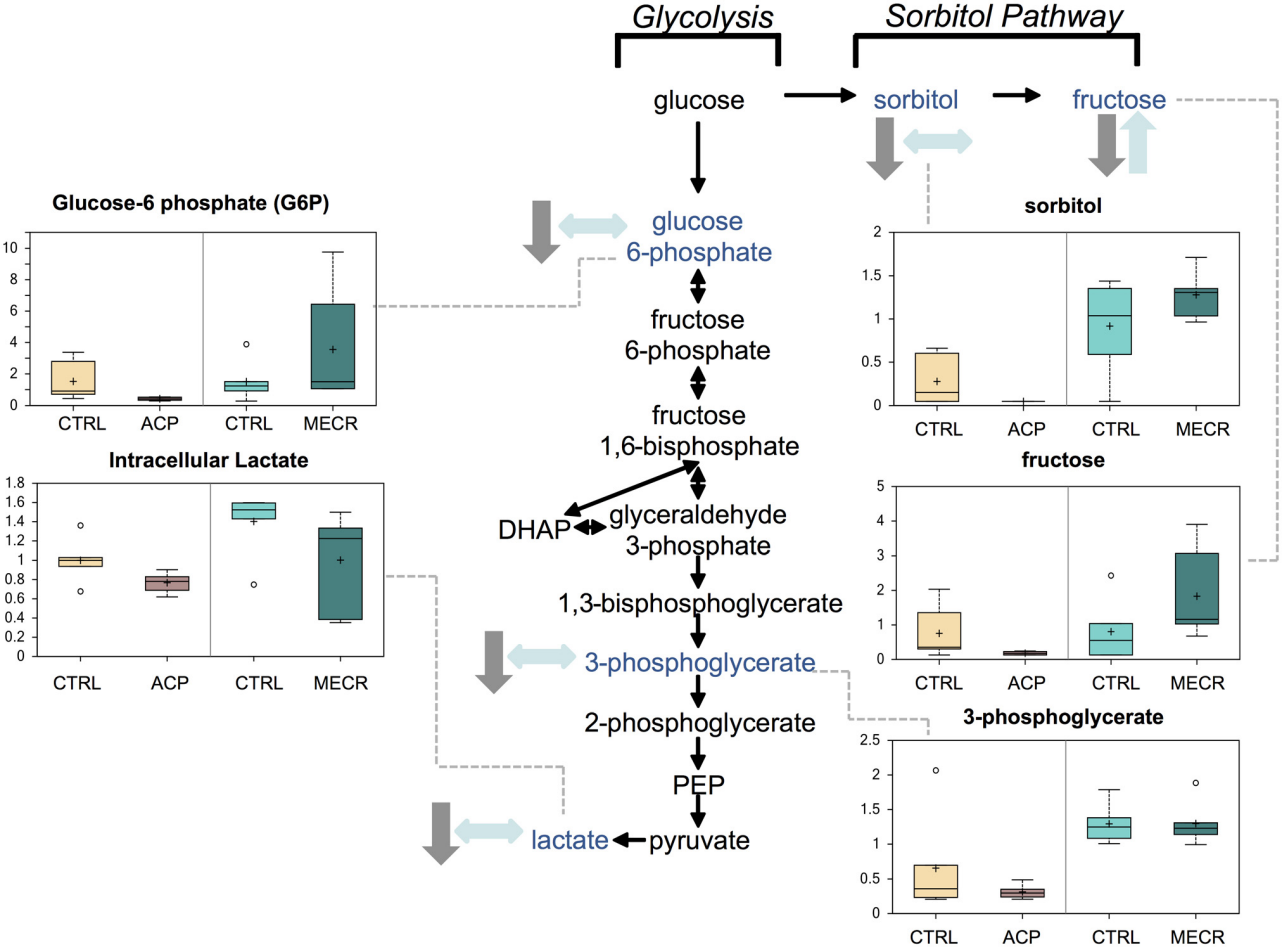
Glycolysis and the sorbitol pathway are significantly altered by changes in mtFASII functionality. Levels of metabolites from glycolysis and the sorbitol pathway were analyzed in whole ACP KD and MECR OX cells and their respective controls using metabolomics, which included UHLC/MS/MS and GC/MS, as described in Experimental Procedures. Glycolysis intermediates that were identified are indicated with arrows and a corresponding box plot. Y-axes for box plots indicate scaled intensity, except where indicated otherwise. Direction of metabolite level change compared to control in ACP KD cells is indicated by gray arrows, and direction of metabolite level change relative to control in MECR OX cells is indicated by blue arrows. In box plots, metabolite levels are shown for ACP KD cells, MECR OX cells, and their respective controls. For each box, n = 6, and open circles indicate extreme data points.

**Fig 5:**
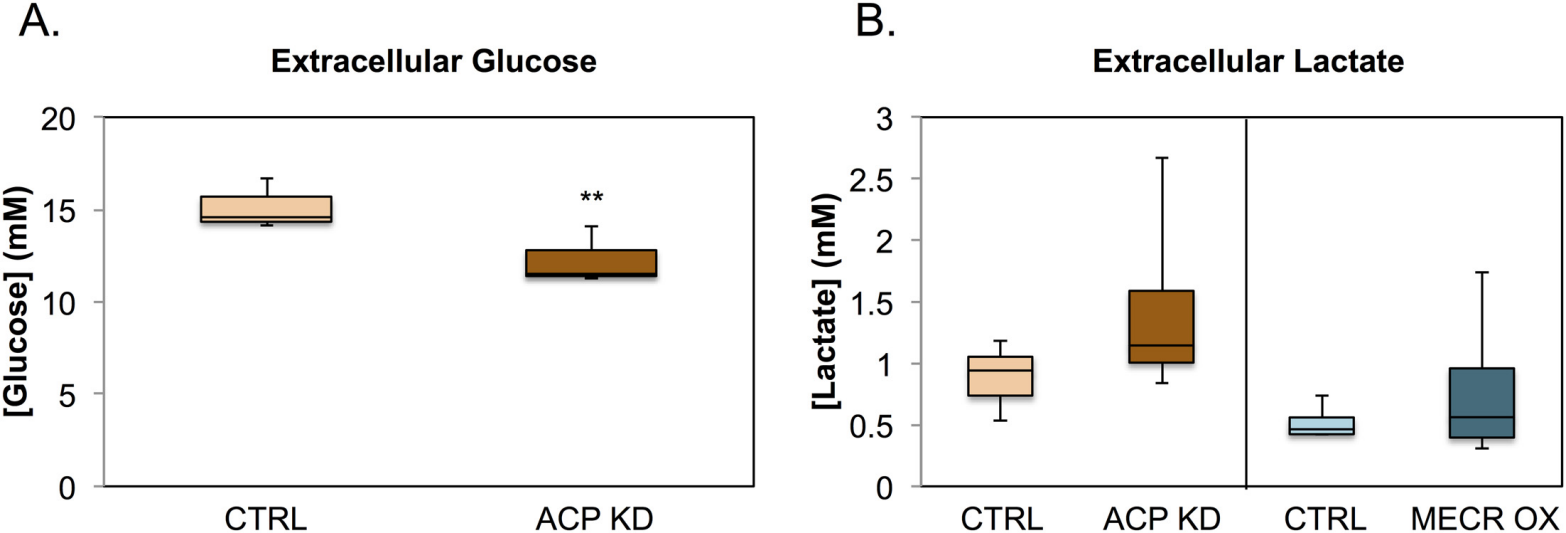
Cell culture media glucose and lactate levels are altered by changes in mtFASII. A) Measurements of cell culture media glucose were taken after 96 h of ACP KD cell culture or after 24 h of MECR OX cell culture, along with their respective controls. Glucose levels were measured in cell culture media using the glucose oxidase method, as described in Experimental Procedures. n = 3, ** indicates p < 0.01. B) Extracellular lactate was measured in cell culture media using a colorimetric lactate assay (Biovision). n = 4. Changes in both conditions are not significant.

Levels of molecules in both the glycolysis and sorbitol pathways were changed as a result of mtFASII dysregulation (Fig 4). Intermediates in the glycolysis pathway, including glucose 6-phosphate and 3-phosphoglycerate, as well as the end product lactate were significantly reduced in ACP KD cells. Intermediates in the sorbitol (or polyol) pathway, including sorbitol and fructose, were at decreased levels in the ACP KD cells, and fructose levels were increased in the MECR OX cells. These results suggest that ACP KD cells are using glycolysis instead of oxidative phosphorylation, and as such are depleting glucose in the medium. To confirm the depletion of glucose by glycolysis in these cells, levels of glucose were measured in the medium after 96 h of knockdown of ACP. Extracellular glucose levels were significantly reduced in ACP KD media (Fig 5).

### Tricarboxylic acid (TCA) cycle and amino acid anaplerosis

**Fig 6:**
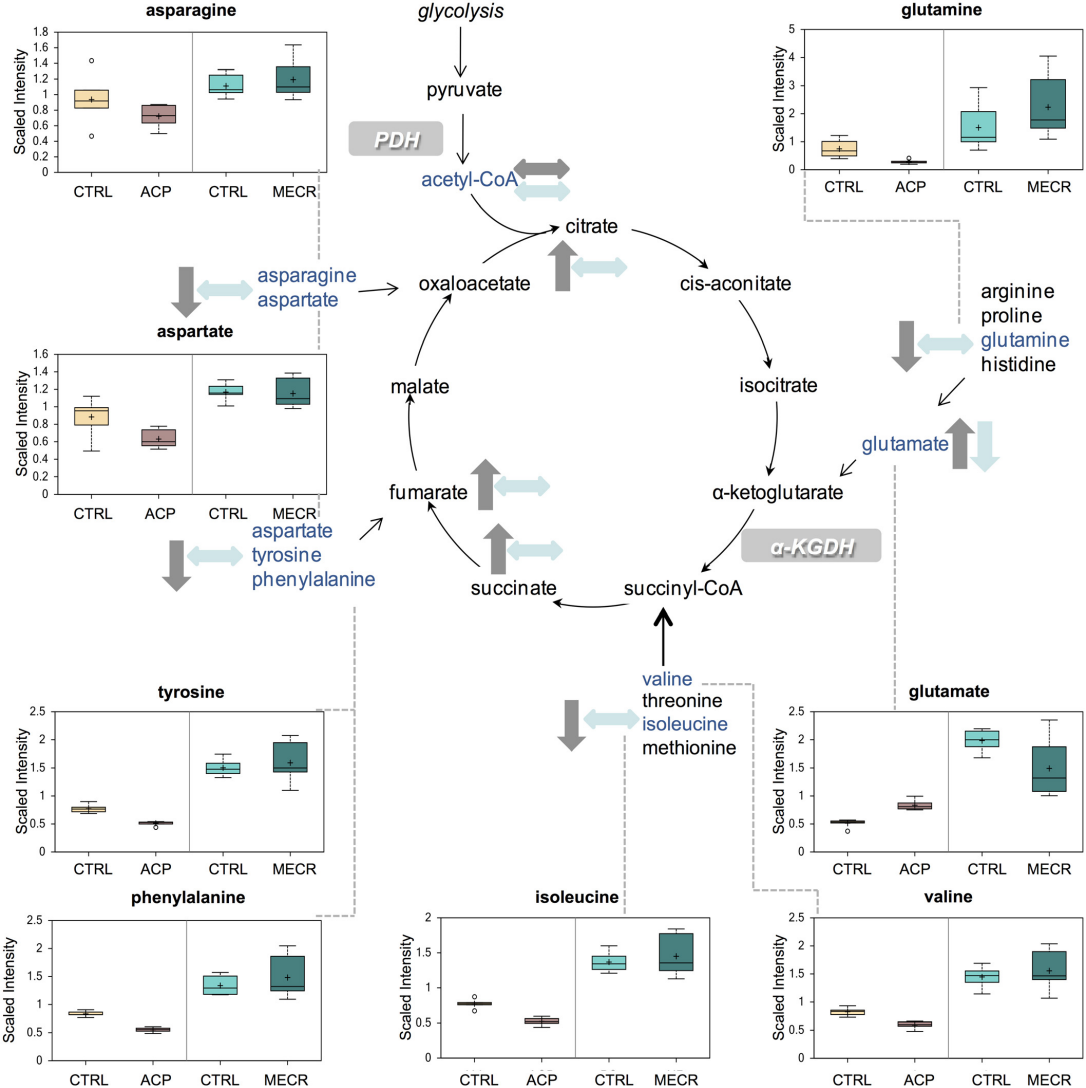
Amino acids used in amino acid anaplerosis are largely depleted in ACP KD cells. Levels of metabolites from amino acid anaplerosis and the TCA cycle were analyzed in whole ACP KD and MECR OX cells and their respective controls using metabolomics, which included UHLC/MS/MS and GC/MS, as described in Experimental Procedures. Amino acid anaplerosis intermediates that were identified are indicated with arrows and a corresponding box plot. Y-axes for box plots indicate scaled intensity. Direction of change in metabolite levels compared to control in ACP KD is indicated by gray arrows, and direction of change of metabolite levels relative to control in MECR OX is indicated by blue arrows. In box plots, metabolite levels are shown for ACP KD cells, MECR OX cells, and their respective controls. For each box, n = 6 and open circles indicate extreme data points.

In ACP KD cells, levels of several TCA cycle intermediates, including citrate, succinate, and fumarate, were significantly elevated. Correspondingly, levels of several amino acids were decreased in ACP KD cells (Fig 6), suggesting that amino acid anaplerosis is compensating for defects in the TCA cycle likely due to loss of mtFASII-related protein lipoylation of TCA cycle enzymes.

### Pentose phosphate pathway

**Fig 7:**
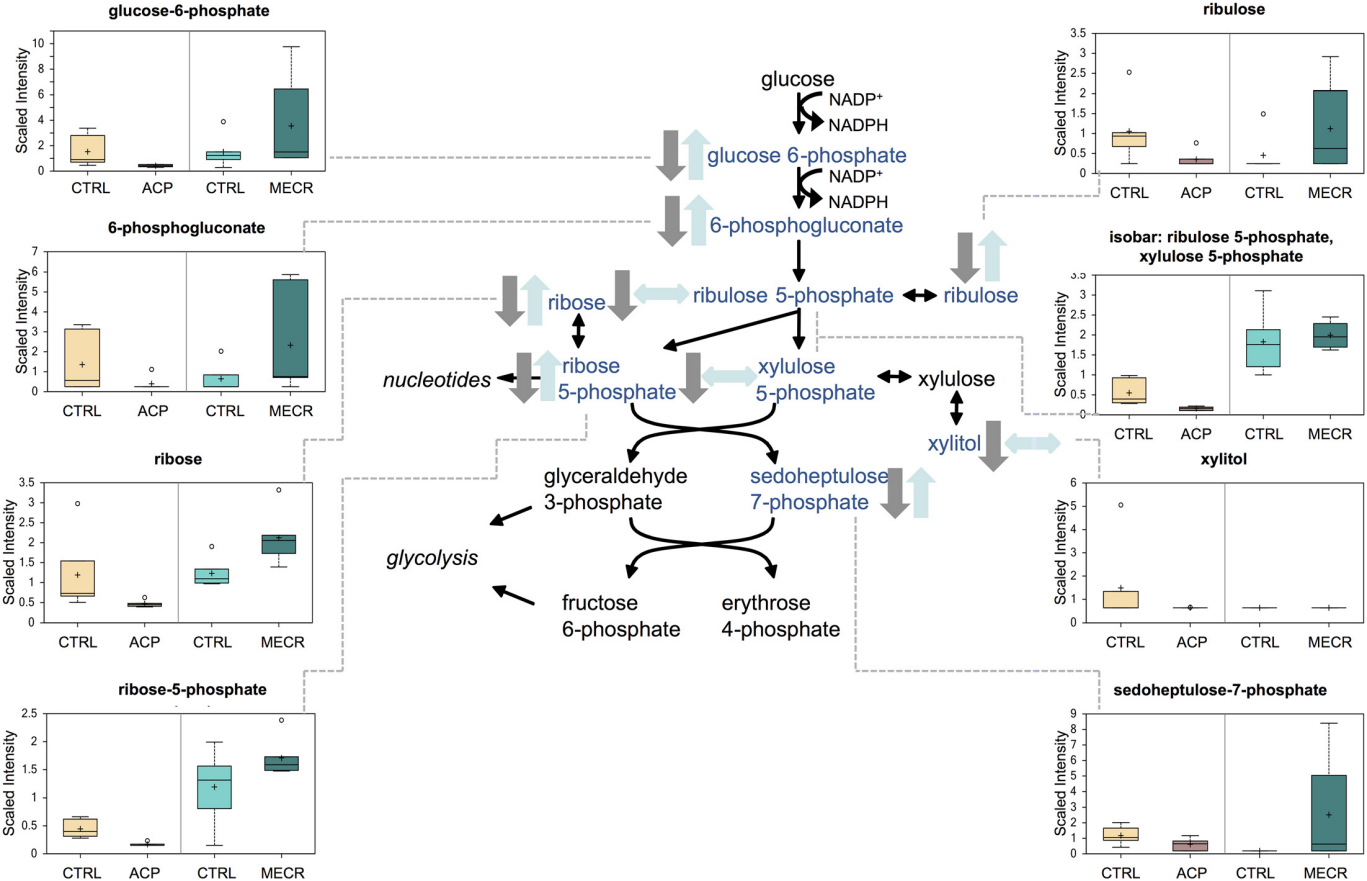
Pentose phosphate pathway metabolites are reduced in ACP KD cells and elevated in MECR OX cells. Levels of metabolites from the pentose phosphate pathway were analyzed in whole ACP KD and MECR OX cells and their respective controls using metabolomics, which included UHLC/MS/MS and GC/MS, as described in Experimental Procedures. Pentose phosphate pathway intermediates that were identified are indicated with arrows and a corresponding box plot. Y-axes for box plots indicate scaled intensity. Direction of metabolite level change compared to control in ACP KD cells is indicated by gray arrows, and direction of metabolite level change relative to control in MECR OX cells is indicated by blue arrows. In box plots, metabolite levels are shown for ACP KD cells, MECR OX cells, and their respective controls. For each box, n = 6 and open circles indicate extreme data points.

Levels of many intermediates in the pentose phosphate pathway (PPP) were decreased in ACP KD cells, and most of these were increased in the MECR OX cells (Fig 7). Like glycolysis, the PPP involves oxidation of glucose, but the pathway is anabolic rather than catabolic. Molecules from both the first part of the pathway that creates reducing equivalents (NADPH), and the second part of the pathway that makes five-carbon sugars were changed in the mtFASII KD and OX cells.

### Redox Status: γ-Glutamyl Cycle

**Fig 8:**
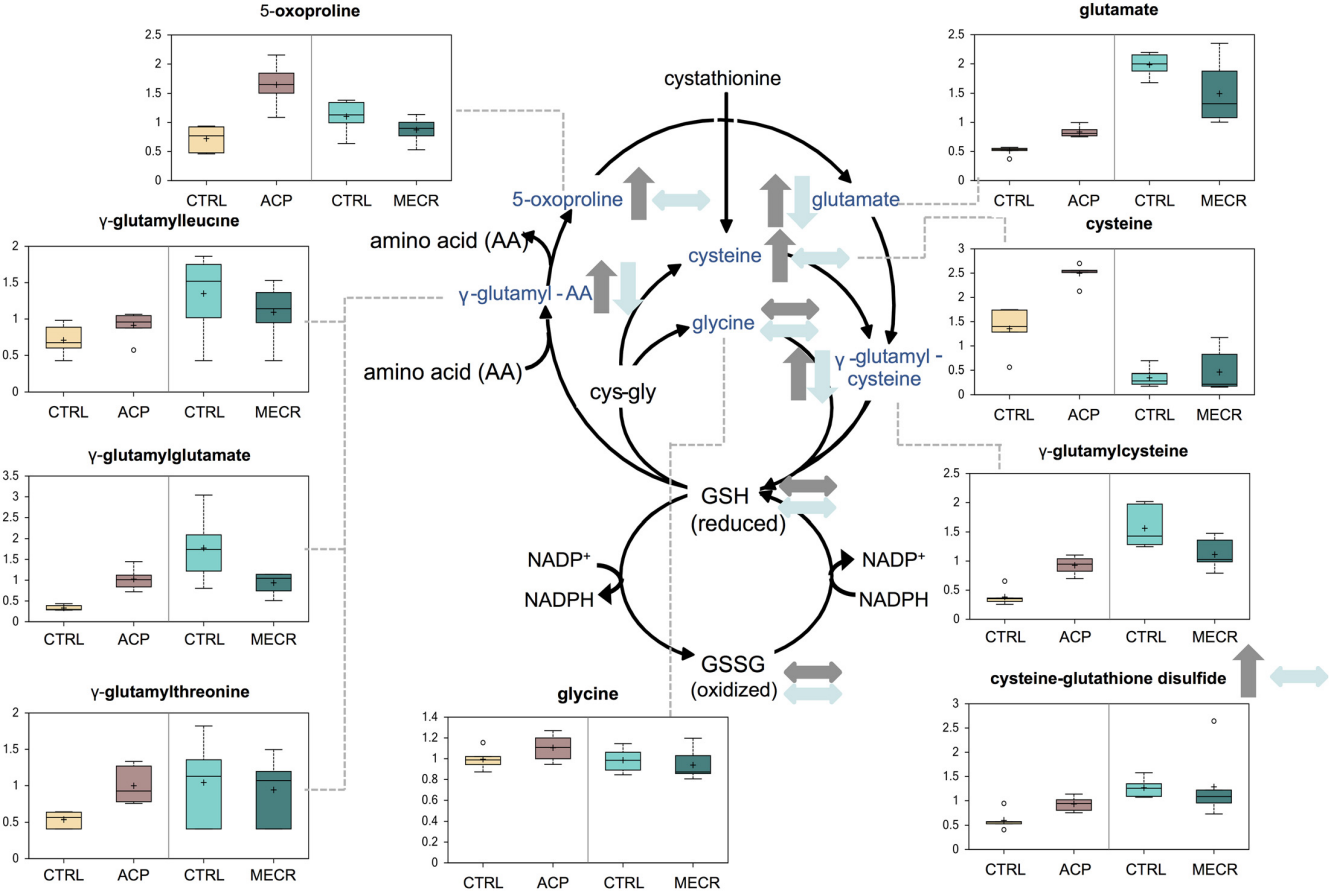
Markers of oxidative stress are altered with changes in mtFASII functionality. Levels of metabolites from the γ-glutamyl cycle were analyzed in whole ACP KD and MECR OX cells and their respective controls using metabolomics, which included UHLC/MS/MS and GC/MS, as described in Experimental Procedures. Gamma-glutamyl cycle intermediates that were identified are indicated with arrows and a corresponding box plot. Y-axes for box plots indicate scaled intensity. Direction of metabolite level change compared to control in ACP KD cells is indicated by gray arrows, and direction of metabolite level change relative to control in MECR OX is indicated by blue arrows. In box plots, metabolite levels are shown for ACP KD cells, MECR OX cells, and their respective controls. For each box, n = 6 and open circles indicate extreme data points.

**Fig 9:**
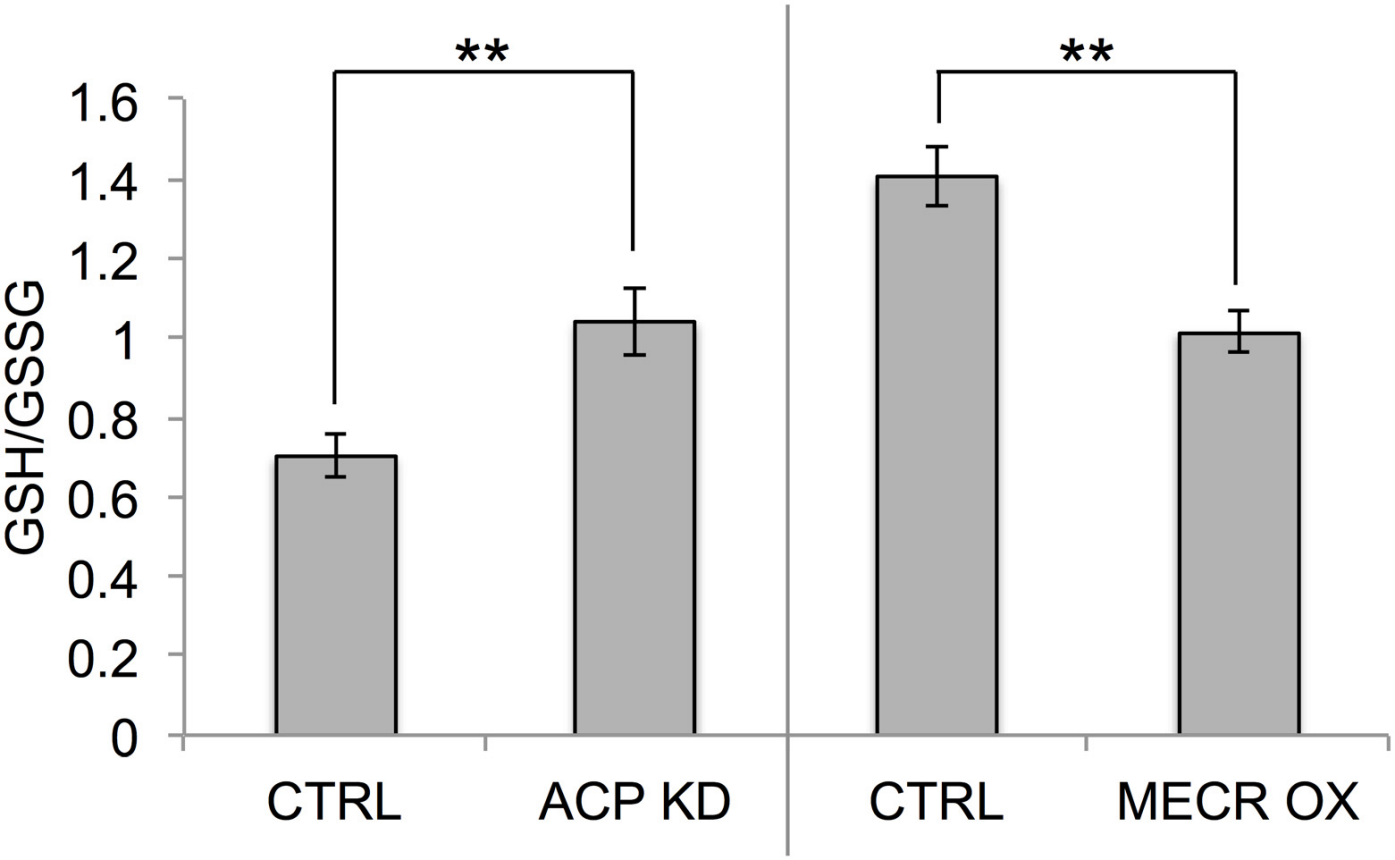
GSH/GSSG ratios are altered by changes in mtFASII function. GSH and GSSG levels in ACP KD cells, MECR OX cells, and their respective controls were determined by metabolomics analysis using UHLC/MS/MS, as described in Experimental Procedures. From these data, GSH/GSSG ratios were calculated. n = 6 and ** p < 0.01.

Many of the intermediates in the γ-glutamyl cycle were also changed in opposite directions in ACP KD and MECR OX cells (Fig 8). Glutamate and γ-glutamyl-linked amino acids, including γ-glutamyl-cysteine, were increased in ACP KD and decreased in MECR OX. In addition, 5-oxoproline, cysteine, and cysteine-glutathione disulfide were all increased in ACP KD cells. These metabolites are intermediates in the synthesis of glutathione, which plays a key role in cellular defenses against oxidative stress. While intermediates in glutathione (GSH) synthesis were increased significantly, levels of reduced GSH were slightly increased in ACP KD cells. Because GSH levels are dictated by a balance between synthesis of reduced GSH and oxidation to glutathione disulfide (GSSG), we also examined the ratio of GSH to GSSG. The GSH/GSSG ratio was significantly different when mtFASII is altered (Fig 9). An increase in the GSH/GSSG ratio in the ACP KD compared to control indicates a more reduced environment, while a decrease in the ratio in MECR OX compared to control cells suggests a more oxidizing environment.

### Polyamine Synthesis

**Fig 10:**
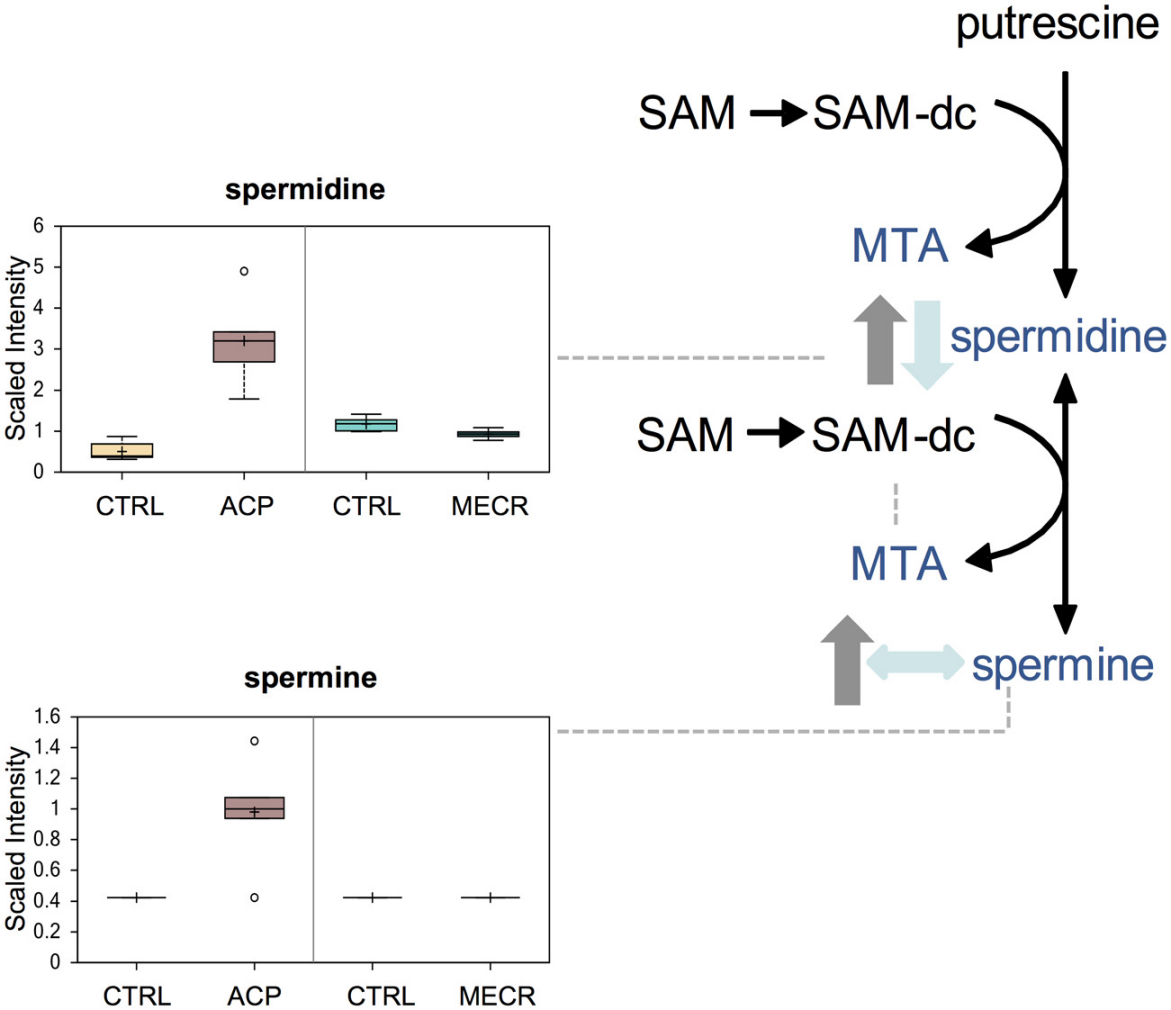
ACP KD results in upregulation of the polyamine synthesis pathway. Levels of metabolites from the polyamine synthesis pathway were analyzed in whole ACP KD and MECR OX cells and their respective controls using metabolomics, which included UHLC/MS/MS and GC/MS, as described in Experimental Procedures. Polyamine synthesis pathway intermediates that were identified are indicated with arrows and a corresponding box plot. Y-axes for box plots indicate scaled intensity. Direction of metabolite level change compared to control in ACP KD cells is indicated by gray arrows, and direction of metabolite level change relative to control in MECR OX cells is indicated by blue arrows. In box plots, metabolite levels are shown for ACP KD cells, MECR OX cells, and their respective controls. For each box, n = 6 and open circles indicate extreme data points.

In ACP KD cells, levels of the polyamines spermidine and spermine were elevated, whereas in MECR OX, spermidine levels were reduced while spermine levels were unchanged (Fig 10).

### Bioactive Lipid Metabolism

**Fig 11:**
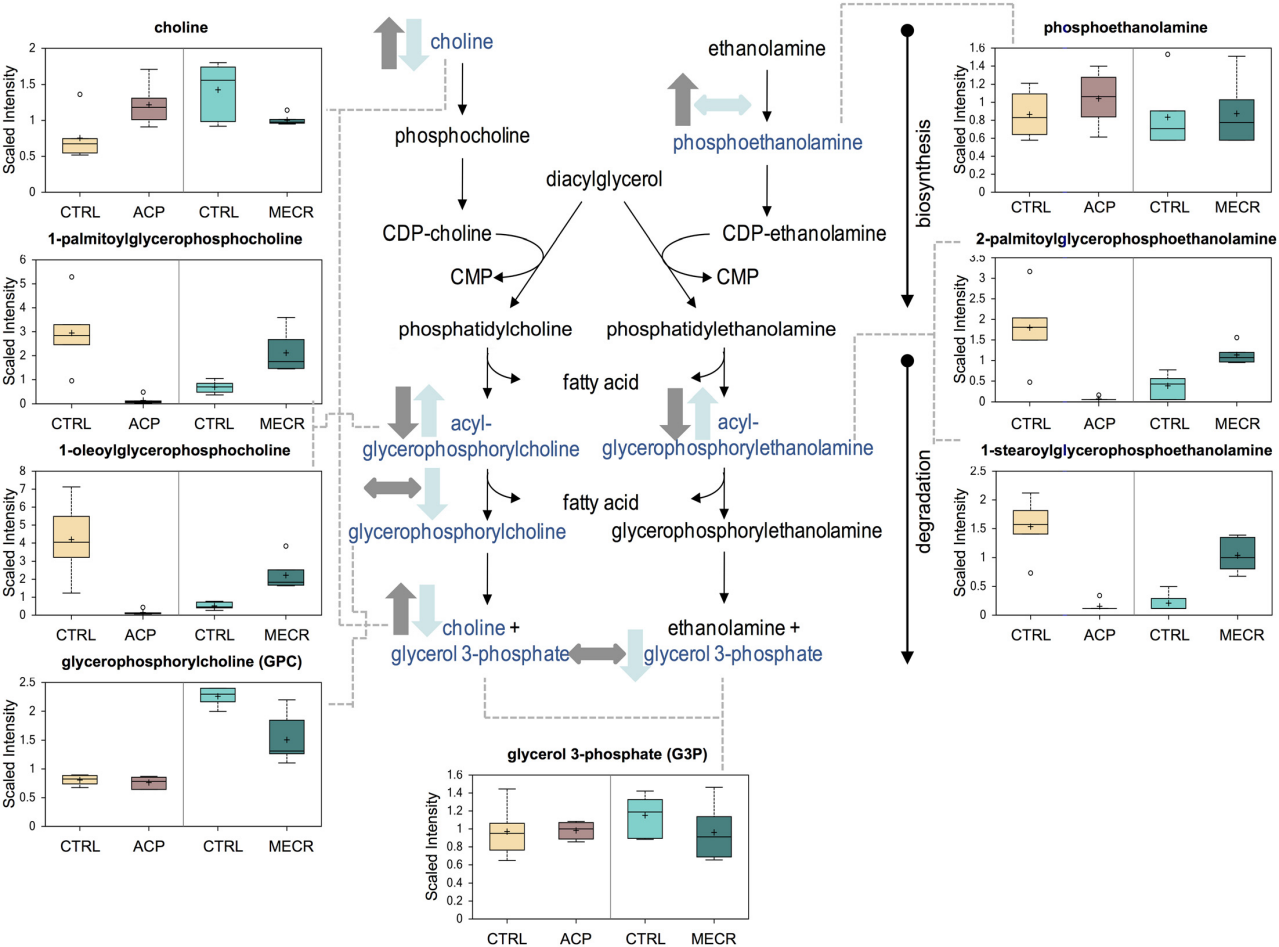
Lipid homeostasis is altered by ACP KD and MECR OX. Levels of metabolites from lipid synthesis and degradation pathways were analyzed in whole ACP KD and MECR OX cells and their respective controls using metabolomics, which included UHLC/MS/MS and GC/MS, as described in Experimental Procedures. Lipid synthesis and degradation intermediates that were identified are indicated with arrows and a corresponding box plot. Y-axes for box plots indicate scaled intensity. Direction of metabolite level change compared to control in ACP KD cells is indicated by gray arrows, and direction of metabolite level change relative to control in MECR OX cells is indicated by blue arrows. In box plots, metabolite levels are shown for ACP KD cells, MECR OX cells, and their respective controls. For each box, n = 6 and open circles indicate extreme data points.

**Fig 12:**
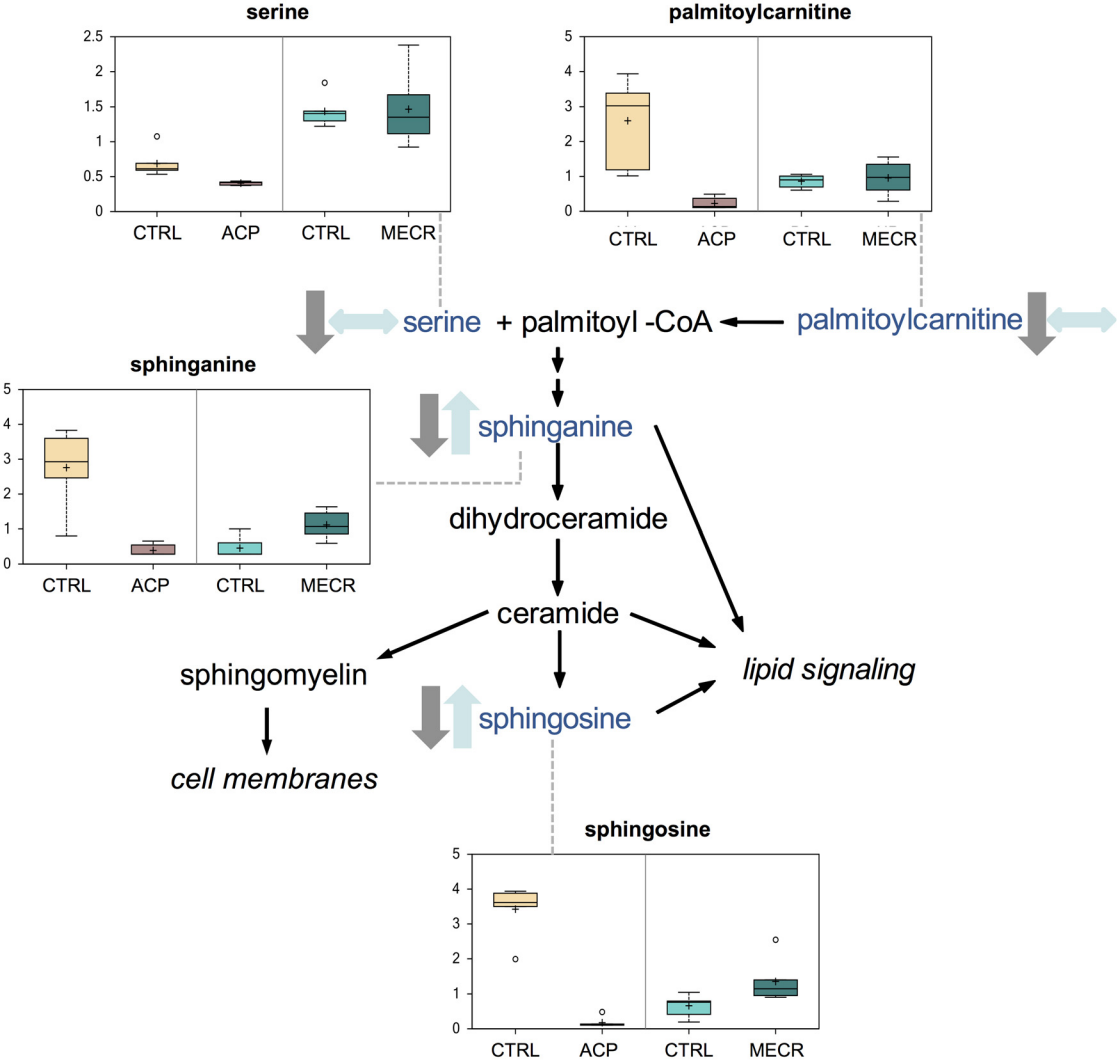
Sphingolipid synthesis metabolites are downregulated in ACP KD and largely upregulated in MECR OX. Levels of metabolites from the sphingolipid synthesis pathway were analyzed in whole ACP KD and MECR OX cells and their respective controls using metabolomics, which included UHLC/MS/MS and GC/MS, as described in Experimental Procedures. Sphingolipid synthesis pathway intermediates that were identified are indicated with arrows and a corresponding box plot. Y-axes for box plots indicate scaled intensity. Direction of metabolite level change compared to control in ACP KD cells is indicated by gray arrows, and direction of metabolite level change relative to control in MECR OX cells is indicated by blue arrows. In box plots, metabolite levels are shown for ACP KD cells, MECR OX cells, and their respective controls. For each box, n = 6 and open circles indicate extreme data points.

**Fig 13:**
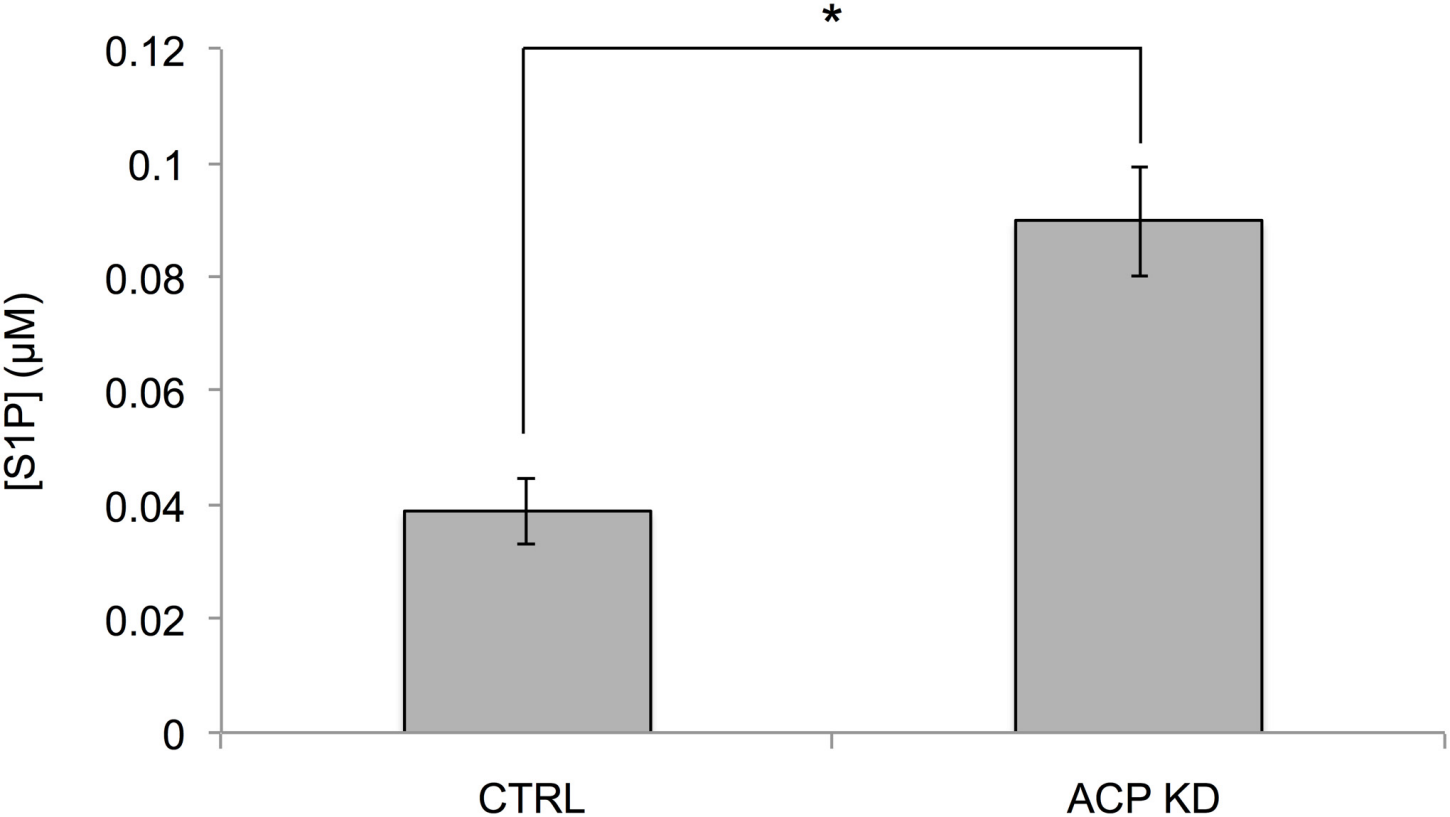
S1P levels are increased in ACP KD cells. S1P levels were measured by ELISA of 40 μg protein from control and ACP KD cells as described in Experimental Procedures. S1P levels are expressed in μM concentrations. n = 3, and * p < 0.05.

ACP KD and MECR OX resulted in significant changes in the levels of specific phospholipids that were not measured in the initial TLC-GC experiments, namely lysophospholipids and sphingolipids. Lysophospholipids measured in this study included acyl-glycerophosphocholines, acyl-glycerophosphoinositols, and acyl-glycerophosphoethanolamines. Several side chain length forms of acyl-glycerophosphocholines and acyl-glycerophosphoethanolamines were changed, but acyl-glycerophosphoinositols were unchanged. These lysophospholipids were strikingly diminished in the cells with mtFASII knockdown, and increased at least two-fold when MECR was overexpressed (Fig 11 and Table 2). Sphingolipid levels were also differentially affected in ACP KD and MECR OX cells. Sphinganine and sphingosine levels were decreased in ACP KD cells, but increased in MECR OX cells (Fig 12). In addition, palmitoylcarnitine, a precursor of the palmitoyl-CoA required for production of sphingolipids, was significantly reduced 11-fold in ACP KD cells. To better characterize sphingosine metabolism in ACP KD cells, sphingosine-1-phosphate (S1P) was measured by ELISA. This bioactive lipid signaling molecule, created by the phosphorylation of sphingosine, was increased over two-fold in ACP KD cells (Fig 13), suggesting that one possible reason that sphingosine is down in ACP KD cells because it is being converted into S1P.

**Table 2:** Significantly altered lysophospholipids.

Lysophospholipid	ACP/Control	MECR/Control
1-palmitoylglycerophosphoethanolamine	1.14	**1.57**
2-palmitoylglycerophosphoethanolamine	**0.04**	**2.96**
2-palmitoleoylglycerophosphoethanolamine	**0.12**	**3.57**
1-stearoylglycerophosphoethanolamine	**0.10**	**4.97**
1-oleoylglycerophosphoethanolamine	**1.26**	2.36
2-oleoylglycerophosphoethanolamine	**0.03**	**2.88**
1-arachidonoylglycerophosphoethanolamine	0.98	1.37
2-arachidonoylglycerophosphoethanolamine	**0.04**	**2.49**
2-docosapentaenoylglycerophosphoethanolamine	**0.04**	**2.46**
2-docosahexaenoylglycerophosphoethanolamine	**0.05**	**2.56**
1-stearoylglycerophosphoglycerol	1.61	1.30
1-myristoylglycerophosphocholine	**0.24**	**3.16**
2-myristoylglycerophosphocholine	**0.06**	**2.25**
1-palmitoylglycerophosphocholine	**0.05**	**3.08**
2-palmitoylglycerophosphocholine	**0.02**	**3.17**
1-palmitoleoylglycerophosphocholine	**0.11**	2.08
2-palmitoleoylglycerophosphocholine	**0.03**	**3.52**
1-stearoylglycerophosphocholine	**0.10**	**2.80**
1-oleoylglycerophosphocholine	**0.04**	**4.33**
2-oleoylglycerophosphocholine	**0.02**	**3.99**
2-linoleoylglycerophosphocholine	**0.28**	**2.80**
2-arachidonoylglycerophosphocholine	**0.09**	**3.19**
2-docosapentaenoylglycerophosphocholine	**0.16**	**3.38**
2-docosahexaenoylglycerophosphocholine	**0.21**	1.59
1-palmitoylglycerophosphoinositol	1.50	2.60
1-stearoylglycerophosphoinositol	1.38	2.18
1-oleoylglycerophosphoinositol	1.67	2.23
2-oleoylglycerophosphoinositol	1.47	2.19
1-palmitoylplasmenylethanolamine	1.05	1.17

Lysophospholipid levels were analyzed in whole ACP KD and MECR OX cells and their respective controls using metabolomics, which included UHLC/MS/MS and GC/MS, as described in Experimental Procedures. All lysophospholipids identified are presented with their corresponding levels in ACP KD or MECR OX cells, each normalized to its own control. Numbers in bold represent significant upregulation or downregulation compared to control cells. Metabolite levels were deemed significantly different from control when p < 0.05.

## Discussion

While it is clear that mitochondria can synthesize fatty acids, questions remain about how disruption of the mtFASII pathway causes the extensive array of observed phenotypes. A lack of change in the fatty acid profiles of polar lipids, triglycerides, and free fatty acids in ACP KD mitochondria indicates that the mtFASII pathway is likely not a major contributor to mitochondrial fatty acid composition. If the mtFASII pathway is not an important contributor to mitochondrial membrane lipid composition, we reasoned that perhaps the pathway plays other roles in the cell. For this reason, we took a broad approach, using metabolomics in whole cells with altered mtFASII function to investigate the roles of the mtFASII pathway in the cell. Pathway analysis of the biochemicals changed upon altering mtFASII function links the mtFASII pathway to a general switch in energy metabolism, redox state, polyamine levels, and bioactive lipid levels.

Manipulation of mtFASII function elicits changes in several pathways that reveal a common theme of altered glucose utilization. Intermediates in glycolysis, the sorbitol pathway, amino acid anaplerosis, and the pentose phosphate pathway are all changed in a way that suggests increased glucose utilization with knockdown of the pathway, and decreased glucose utilization with increased function of the pathway. Correspondingly, we find that extracellular glucose is decreased and extracellular lactate is increased in ACP KD, both of which point to increased glycolysis. This is consistent with previous work, as knockdown of any mtFASII component in yeast or mammalian cells leads to defects in oxidative phosphorylation and subsequent increased reliance on glycolysis [12, 15, 17, 35]

Another glucose-responsive pathway, the TCA cycle, is also altered by changes in mtFASII function. The intermediates in the TCA cycle, however, are increased in the ACP KD, and levels of acetyl-CoA remain the same. Since a lack of mtFASII would be expected to interfere with the TCA cycle by decreased lipoylation of pyruvate dehydrogenase and α-ketoglutarate dehydrogenase, the increased levels of these intermediates suggests that a compensatory mechanism is in place. Amino acid stores are decreased in the ACP KD, likely indicating that increased amino acid anaplerosis replenishes TCA cycle metabolite levels [36] in response to depleted glucose levels or dysfunctional TCA cycle enzymes.

Changes in all of the above-mentioned glucose responsive pathways are limited mainly to decreased intermediates in the ACP KD cells with very few changes observed in the MECR OX cells. Pentose phosphate pathway (PPP) intermediates, however, are upregulated in the MECR OX cells as well as decreased in the ACP KD cells. The PPP generates much of the NADPH in the cell, and is an essential source of metabolites important to several biosynthetic pathways [37]. The rate-limiting enzyme of PPP, glucose-6-phosphate dehydrogenase (G6PD), exhibits increased activity with reductions in the NADPH/NADP ratio [37, 38]. In the MECR OX cells, enhanced mtFASII function could increase NADPH consumption, lowering the NADPH/NADP ratio, thereby increasing G6PD activity and PPP flux. Though mtFASII is not the cell’s main consumer of NADPH, ACP KD could elevate NADPH levels, which would increase the NADPH/NADP ratio, causing a decrease in G6PD activity.

Gamma-glutamyl cycle intermediates show an opposite pattern to the PPP intermediates, and are increased in the ACP KD cells and decreased in the MECR OX cells. In general, increases in γ-glutamyl cycle metabolites are correlated with oxidative stress, suggesting increased oxidative stress in the ACP KD cells. In MECR OX cells, however, several GSH synthesis metabolites are at decreased levels, indicating that the cells have decreased oxidative stress, perhaps due to increased levels of the antioxidant and mtFASII product lipoic acid. GSH and GSSG, the most abundant markers of the pathway, appear relatively unchanged in ACP KD cells, although it is possible that changes in these molecules were masked by their conversion to other molecular species or their export from the cell [39–41]. The closely related molecule, cysteine-glutathione disulfide is retained in the cell, its levels are proportional to redox status, [39, 42, 43] and it is increased in ACP KD cells. Surprisingly, the most widely used indicator of oxidative stress, the GSH/GSSG ratio, is higher in ACP KD cells, indicating a more reduced environment, and lower in MECR OX cells, suggesting a more oxidizing environment. This suggests that although the γ-glutamyl cycle is upregulated by mtFASII dysfunction, the increased pools of NADPH caused by decreases in mtFASII activity promote the reduction of GSSG to GSH, creating a more reducing environment, and that the opposite is happening with improved mtFASII function

The levels of the polyamines spermine and spermidine are increased in ACP KD cells, and spermidine is decreased in MECR OX cells. An increase in ROS could be responsible for increased polyamine levels in ACP KD cells. During oxidative stress polyamines prevent DNA nicking and protein carbonylation, enhancing cell survival [44, 45]. Ornithine decarboxylase (ODC) and spermidine/spermine-N^1^-acetyltransferase (SSAT), enzymes involved in the synthesis and degradation of polyamines, respectively, are upregulated in both expression and activity during oxidative stress [44, 46]. The upregulation of ODC, however, outpaces that of SSAT, resulting in polyamine accumulation [44, 46]. Alternatively, changes in polyamine levels could be in response to mitochondrial RNA processing defects associated with mtFASII dysfunction [6, 18], since polyamines bind to RNA and are important in RNA processing and expression [47, 48].

The most striking changes observed in the ACP KD and MECR OX were in bioactive lipid levels, including lysophospholipids and sphingolipids. Levels of both classes of bioactive lipids were directly correlated with mtFASII activity, with 20 of 31 identified species showing significant differences in both directions of mtFASII change, and an additional four species showing significant changes in one condition. Lysophospholipids that responded to changes in mtFASII function are species of phosphatidylethanolamine and phosphatidylcholine containing a single acyl chain. Lysophospholipids are often the products of phospholipid remodeling or breakdown, but can also be synthesized directly in the cytosol via acylation of glycerol-3-phosphate by glycerol-3-phosphate acyltransferase, or in the mitochondria by mitochondrial glycerol-3-phosphate acyltransferase [49–51].

The mtFASII pathway is known to affect lysophospholipid levels as ACP knockdown in the fungi *N*. *crassa* resulted in elevated mitochondrial membrane lysophospholipid levels [14] ACP knockdown in the yeast *S*. *cerevisiae*, however, had no effect on lysophospholipid levels [14]. The loss of lysophospholipids in ACP KD cells, together with increased choline and phosphoethanolamine levels, suggest that the canonical pathway for generation of the phospholipids phosphatidylethanolamine and phosphatidylcholine is not optimally functional. Suppression of the PPP in ACP KD cells would lead to a reduction in PPP-mediated synthesis of cytidine diphosphate (CDP), a molecule used in the canonical phospholipid synthesis pathway. ACP KD-related suppression of the PPP would decrease CDP levels. Hence, loss of mtFASII might lead to loss of the canonical pathway for synthesis of phosphatidylethanolamine and phosphatidylcholine, and the compensatory conversion of lysophospholipids into phospholipids. This correlates also with the increase in both PPP and lysophospholipids in the MECR OX cells. Taken together, these data suggest a model where mtFASII function alters the NADPH/NADP ratio, which alters flux through the PPP, rendering downstream effects on CDP levels, the canonical phospholipid synthesis pathway, and lysophospholipid levels.

Sphingolipids, the second class of bioactive lipids for which samples were examined, are lipids derived from the condensation of serine and palmitoyl-CoA and are characterized by an 18-carbon backbone. Our data indicate that levels of sphingolipids and their precursors are reduced in ACP KD and increased in MECR OX. Sphingolipids are important determinants of cell survival, as ceramides and sphingosine promote growth arrest and apoptosis, while S1P promotes proliferation and survival [52–57]. Sphingosine and S1P are interconvertible through specific kinases and phosphatases; because of this interconvertibility and opposite signaling functions, the relative levels of sphingosine and S1P are important markers of cell or organelle health. In ACP KD cells, sphingosine levels are decreased, while S1P levels are increased. These changes could be a result of the cell compensating for damage caused by respiratory deficiency and increased oxidative stress, but further analysis is necessary to understand the mechanisms underlying changes in sphingolipid levels under altered mtFAS II function.

The role mtFASII plays in the mitochondria and the cell is not entirely clear. The present study provides insight into the specific molecules and pathways affected by changes in mtFASII function. Our data regarding metabolic states in cells with reduced mtFASII function is consistent with previously published research, and provides an in-depth look at the root of mitochondrial dysfunction upon loss of ACP. The observation that there is a connection between mtFASII function and bioactive lipid levels is intriguing, and the mechanism behind this relationship awaits further investigation.

## Supporting Information

S1 Fig Full metabolomics dataset for analysis of ACP KD and MECR OX cells. (XLSX)
